# The Effect of Cannabidiol on Nociceptive Behaviour and the Endocannabinoid System in an Incisional Wound Model

**DOI:** 10.3390/ph19010043

**Published:** 2025-12-24

**Authors:** Maria C. Redmond, Catherine R. Healy, Mary Hopkins, Rosmara Infantino, Georgina Gethin, Abhay Pandit, David P. Finn

**Affiliations:** 1 Pharmacology and Therapeutics, School of Pharmacy and Medical Sciences, Institute for Health Discovery and Innovation, University of Galway, H91 W5P7 Galway City, Ireland; m.redmond8@universityofgalway.ie (M.C.R.);; 2 Galway Neuroscience Centre, University of Galway, H91 TK33 Galway City, Ireland; 3 Centre for Pain Research, University of Galway, H91 TK33 Galway City, Ireland; 4 CÚRAM, Research Ireland Centre for Medical Devices, University of Galway, H91 W2TY Galway City, Ireland; georgina.gethin@universityofgalway.ie (G.G.); abhay.pandit@universityofgalway.ie (A.P.); 5 School of Nursing and Midwifery, University of Galway, H91 E3YV Galway City, Ireland; 6 Alliance for Research and Innovation in Wounds, University of Galway, H91 TK33 Galway City, Ireland

**Keywords:** cannabidiol, wound, pain, endocannabinoid system

## Abstract

**Background/Objectives:** Wound-related pain is a common, yet inadequately managed condition, and new therapeutic strategies are warranted. Limited data suggests that phytocannabinoids and cannabis may alleviate wound-related pain; however, further studies are required. This study investigated the effects of systemic administration of cannabidiol (CBD) on nociceptive behaviour following dorsum incision and on the endocannabinoid system. **Methods**: Male Sprague-Dawley rats (150–200 g on arrival, *n* = 9/group) underwent a 1.2 cm incision on the hairy skin of the dorsum or sham procedure. Back and hind paw mechanical withdrawal thresholds were assessed at baseline and post-surgery/sham days (PSDs) 1, 4, 7, and 8 using manual and electronic von Frey tests, respectively. On PSD 8, the effect of a single acute administration of CBD (3, 10, or 30 mg/kg, i.p.) on mechanical hypersensitivity in the dorsum and hind paws was assessed. The levels of endocannabinoids and *N*-acylethanolamines in the plasma and discrete brain regions following CBD administration were analysed. **Results**: Robust mechanical hypersensitivity was evident in the dorsum and hind paws following the incision. CBD (3 mg/kg) partially attenuated primary mechanical hypersensitivity in the dorsum, in a site- and dose-specific manner. CBD had no effect on secondary mechanical hypersensitivity. CBD did not alter the levels of endocannabinoids or *N*-acylethanolamines, but in rats that received CBD (3 mg/kg), levels of 2-AG were lower in the contralateral amygdala and levels of AEA were higher in the contralateral lumbar spinal cord, compared to the ipsilateral sides. **Conclusions**: These data provide evidence for antinociceptive effects of CBD in a model of incisional wound-related pain. Further research on CBD’s mechanism(s) of action is warranted. The potential antinociceptive effects of other phytocannabinoids in this model should also be investigated.

## 1. Introduction

Wound-related pain is a major burden, with 31–58% of people who undergo surgery experiencing moderate to severe acute postsurgical pain after hospital discharge [1]. Inadequate pain management after surgery not only increases the risk of postsurgical complications, but the intensity of acute post-surgical pain is a risk factor for the development of chronic postsurgical pain [2,3]. Appropriate pain management strategies and the development of effective therapies for postsurgical pain are unmet clinical needs that must be addressed.

Cannabidiol (CBD) is a phytocannabinoid found in *Cannabis sativa* L. [4]. CBD interacts with many molecular targets, including several members of the transient receptor potential (TRP) ion channel family and the nuclear receptor peroxisome proliferator-activated receptor gamma (PPAR-γ) [5,6]. It has also been reported to act as an indirect agonist of the 5-HT_1A_ receptor and modulate the endocannabinoid system as a negative allosteric modulator of cannabinoid receptors, CB_1_ and CB_2_ [7,8,9].

The antinociceptive effects of CBD have been investigated in several rat pain models. Oral and transdermal administration of CBD has been shown to attenuate both mechanical and heat hypersensitivity in carrageenan-induced and complete Freund’s adjuvant (CFA) models of inflammatory pain [10,11,12,13]. However, pretreatment with CBD had no effect on nociceptive behaviour in the formalin model [14]. Oral and systemic administration of CBD attenuates mechanical, cold, and heat hypersensitivity in neuropathic pain models, including chronic constriction injury (CCI), spared nerve injury (SNI), and streptozotocin-induced neuropathic pain [15,16,17]. The effects of CBD have also been investigated in the paw incision model of postsurgical pain. Three and 10 mg/kg i.p. doses of CBD attenuated mechanical hypersensitivity following paw incision in both male and female rats [18,19]. Time-course data from these studies indicate a maximal decrease in mechanical hypersensitivity 60 min after CBD administration. However, the antinociceptive effects of CBD have not been assessed in the back hairy skin incision model of incisional wound-related pain [20]. The location of the wound on the hairy skin of the dorsum compared to the glabrous skin in the paw incision model and the addition of blunt dissection around the wound may represent a more translationally relevant model of wound-related pain in which the therapeutic potential of compounds can be assessed. It more closely resembles the situation of clinical surgery and results in more persistent mechanical hypersensitivity around the site of the wound [21].

Antagonist studies have indicated that the antinociceptive effects of CBD are independent of activity at CB_1_ and CB_2_ receptors [12,22]. Evidence from these studies suggests that the antinociceptive effects of CBD in pain models are primarily mediated by TRPV1. The role of the 5-HT_1A_ receptor in the antinociceptive effects of CBD in neuropathic pain has also been suggested, with a 5-HT_1A_ receptor antagonist partially reversing the effect of CBD on mechanical hypersensitivity in the SNI model [16]. However, CBD may indirectly target the endocannabinoid system, as it has been shown to increase the levels of the endocannabinoid anandamide in humans [23]. Agonism of PPARγ by CBD alleviated inflammation in a rat model of rheumatoid arthritis, indicating that PPARγ may also be involved in CBD’s antinociceptive effects [24]. Furthermore, the CBD analogue PECD-101 prevented the onset of mechanical and cold hypersensitivity in a mouse model of chemotherapy-induced neuropathic pain, an effect which was blocked by a PPARγ antagonist [25]. This evidence indicates that the antinociceptive effects of CBD, an agonist of PPARγ, may be partially mediated via this receptor.

The aims of the present study were to investigate the effects of acute, systemic administration of CBD on nociceptive behaviour following dorsum incision in male Sprague Dawley rats, and investigate the effect of CBD on the levels of endocannabinoids (2-arachydonyl glycerol [2-AG] and anandamide [AEA]) and related *N*-acylethanolamines (*N*-palmitoylethanolamide [PEA] and *N*-oleoylethanolamide [OEA]), in the plasma, spinal cord, and key brain regions related to nociception. It also aimed to investigate the effect of CBD on the expression of select receptor targets of CBD in the spinal cord and discrete brain regions related to nociception.

## 2. Results

### 2.1. Development of Mechanical Hypersensitivity in the Dorsum and Hind Paws Following Dorsum Incisional Wound Creation

**Figure 1:**
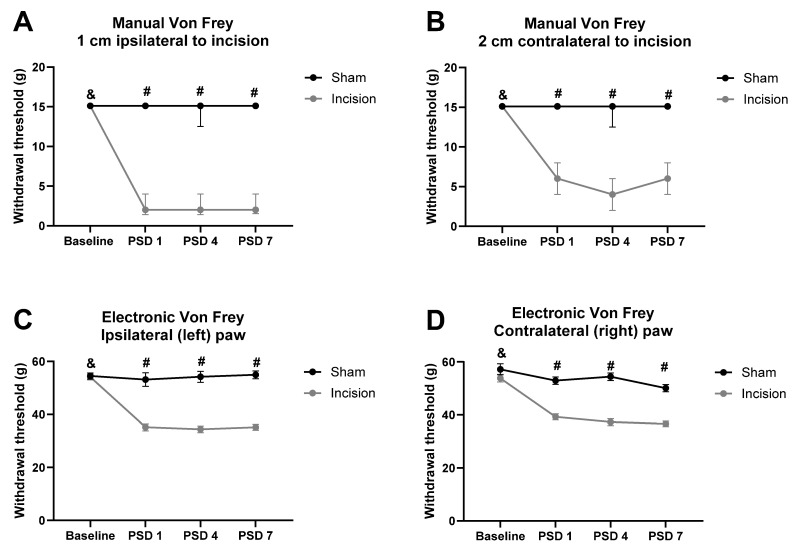
Effect of dorsum incision on mechanical withdrawal thresholds at (**A**) 1 cm ipsilateral to the incision, (**B**) 2 cm contralateral to the incision, (**C**) ipsilateral hind paw, and (**D**) contralateral hind paw. Data are expressed as median ± interquartile range (IQR) (A and B) or mean ± standard error of the mean (SEM) (C and D), *n* = 9 sham, *n* = 36 incision. # *p* < 0.001 sham vs. incision. & *p* < 0.001, incision baseline vs. PSDs 1, 4, and 7. PSD: post-surgery/sham day.

Friedman’s test revealed significant differences in the mechanical withdrawal threshold at 1 cm ipsilateral to the incision χ^2^(5) = 148.140 (*p* < 0.0001). Mann–Whitney U test with Bonferroni–Holm correction revealed a significantly lower mechanical withdrawal threshold at 1 cm ipsilateral to the incision in the incised rats compared to the sham rats at post-surgery/sham days (PSDs) 1, 4, and 7 (*p* < 0.001), as shown in Figure 1A. There were no significant between-group differences in the mechanical withdrawal threshold at 1 cm ipsilateral to the incision at baseline. Wilcoxon’s test with Bonferroni–Holm correction revealed a significantly lower mechanical withdrawal threshold at 1 cm ipsilateral to the incision in rats on PSDs 1, 4 and 7, compared to their baseline (*p* < 0.001). There were no significant differences in mechanical withdrawal threshold at 1 cm ipsilateral to the incision for sham rats compared to their baseline at any time point post-sham procedure, as shown in Figure 1A.

Friedman’s test revealed a significant difference in the mechanical withdrawal threshold at 2 cm contralateral to the incision χ^2^(5) = 153.460 (*p* < 0.001). Mann–Whitney U test with Bonferroni–Holm correction revealed a significantly lower mechanical withdrawal threshold at 2 cm contralateral to the incision in the incised rats compared to the sham rats at PSDs 1, 4, and 7 (*p* < 0.001), as shown in Figure 1B. There were no significant between-group differences in the mechanical withdrawal threshold at 2 cm contralateral to the incision at baseline. Wilcoxon’s test with Bonferroni–Holm correction revealed a significantly lower mechanical withdrawal threshold at 2 cm contralateral to the incision in incision rats on PSDs 1, 4, and 7, compared to baseline (*p* < 0.001). There were no significant differences in the mechanical withdrawal threshold at 2 cm contralateral to the incision for sham rats compared to baseline at any time point post-sham procedure, as shown in Figure 1B.

One-way repeated measures ANOVA on the data prior to drug administration on PSD 8 revealed a significant effect of time (F_(3, 129)_ = 17.610, *p* < 0.0001), time × procedure interaction (F_(3, 129)_ = 16.270, *p* < 0.0001), and procedure (F_(3, 129)_ = 83.241, *p* < 0.0001) on ipsilateral hind paw withdrawal threshold. Independent samples t-test with Bonferroni–Holm correction revealed a significantly lower paw withdrawal threshold in incision rats compared to shams on PSDs 1, 4, and 7 (*p* < 0.001), as shown in Figure 1C. There were no significant between-group differences in the ipsilateral paw withdrawal threshold at baseline. Paired sample t-test with Bonferroni–Holm correction revealed a significantly lower ipsilateral paw withdrawal threshold in incision rats on PSDs 1, 4, and 7, compared to their baseline (*p* < 0.001). There were no significant differences in the ipsilateral paw withdrawal threshold in sham rats compared with their baseline at any time point post-sham procedure, as shown in Figure 1C.

One-way repeated measures ANOVA on the data prior to drug administration on PSD 8 revealed a significant effect of time (F_(3, 129)_ = 2.442, *p* < 0.0001), time × procedure interaction (F_(3, 129)_ = 6.253, *p* < 0.0001), and procedure (F_(3, 129)_ = 50.290, *p* < 0.0001) on contralateral hind paw withdrawal threshold. Independent samples t-test with Bonferroni–Holm correction revealed a significantly lower paw withdrawal threshold in the incision group compared to the sham group on PSDs 1, 4, and 7 (*p* < 0.001), as shown in Figure 1D. There were no significant between-group differences in the contralateral paw withdrawal threshold at baseline. Paired sample *t*-test with Bonferroni–Holm correction revealed a significantly lower contralateral paw withdrawal threshold in incision rats on PSDs 1, 4, and 7, compared to their baseline (*p* < 0.001). There were no significant differences in the contralateral paw withdrawal threshold in sham rats compared with their baseline at any time point post-sham procedure, as shown in Figure 1D.

### 2.2. CBD Partially Attenuates Primary Mechanical Hypersensitivity Following Dorsum Incision

On PSD 8, CBD (3, 10, or 30 mg/kg i.p.) or vehicle (ethanol–Cremophor–saline, 1:1:18) was administered to incision groups 60 min before mechanical hypersensitivity was assessed in the dorsum and paws. The sham group received vehicle only.

**Figure 2:**
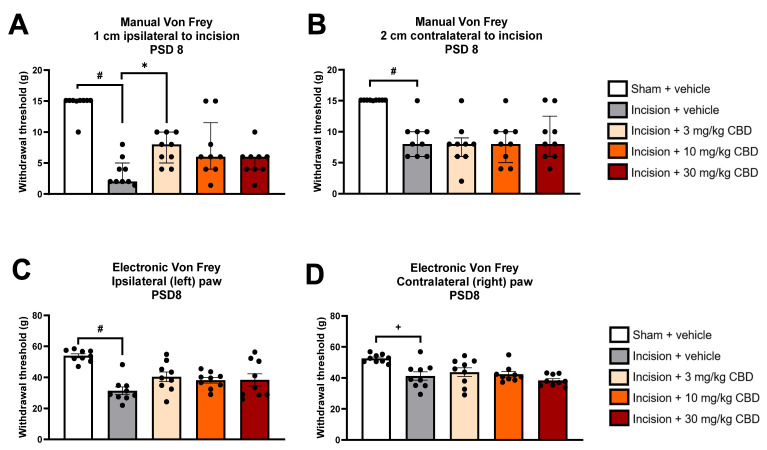
Effect of vehicle or CBD administration on mechanical withdrawal thresholds at (**A**) 1 cm ipsilateral to the incision, (**B**) 2 cm contralateral to the incision, (**C**) ipsilateral hind paw and (**D**) contralateral hind paw 60 min post-administration. Data are expressed as median ± I.Q.R (**A**,**B**) or mean ± S.E.M (**C**,**D**), *n* = 9 per group. # *p* < 0.001 sham vehicle vs. incision vehicle, + *p* < 0.01 sham vehicle vs. incision vehicle, * *p* < 0.01 incision vehicle vs. incision-CBD (3 mg/kg).

Kruskal–Wallis test revealed significant differences in the mechanical withdrawal threshold at 1 cm ipsilateral to the incision 60 min following drug administration on PSD 8 χ^2^(4) = 26.100 (*p* < 0.001). Mann–Whitney U test with Bonferroni–Holm correction revealed a significantly lower mechanical withdrawal threshold at 1 cm ipsilateral to the incision in incision-vehicle rats vs. sham-vehicle rats (*p* < 0.001). Mann–Whitney U test with Bonferroni–Holm correction revealed there was a significantly higher mechanical withdrawal threshold in incision-CBD (3 mg/kg) vs. incision-vehicle rats (*p* < 0.01), indicating that CBD (3 mg/kg) partially attenuated incision-related mechanical hypersensitivity at 1 cm ipsilateral to the incisional wound, as shown in Figure 2A. Mann–Whitney U test with Bonferroni–Holm correction revealed no significant difference in the mechanical withdrawal threshold at 1 cm ipsilateral to the incision between incision-CBD (10 mg/kg) vs. incision-vehicle rats (*p* = 0.063) or between incision-CBD (30 mg/kg) vs. incision-vehicle rats (*p* = 0.136), indicating that these doses of CBD do not significantly attenuate incision-related mechanical hypersensitivity at 1 cm ipsilateral to the incision 60 min post-administration, as shown in Figure 2A.

Kruskal–Wallis test revealed significant differences in the mechanical withdrawal threshold at 2 cm contralateral to the incision 60 min following drug administration on PSD 8 χ^2^(4) = 20.477 (*p* < 0.001). Mann–Whitney U test with Bonferroni–Holm correction revealed a significantly lower mechanical withdrawal threshold at 2 cm contralateral to the incision in incision-vehicle rats vs. sham-vehicle rats (*p* < 0.001), as shown in Figure 2B. Mann–Whitney U test with Bonferroni–Holm correction revealed no significant difference in the mechanical withdrawal threshold at 2 cm contralateral to the incision between incision-CBD (3 mg/kg) vs. incision-vehicle rats (*p* = 0.605), between incision-CBD (10 mg/kg) vs. incision-vehicle rats (*p* = 0.796), or between incision-CBD (30 mg/kg) vs. incision-vehicle rats (*p* = 0.931), indicating that CBD does not significantly attenuate incision-related mechanical hypersensitivity at 2 cm contralateral to the incision 60 min post-administration, as shown in Figure 2B.

One-way ANOVA revealed a significant effect of treatment (F_(4, 40)_ = 9.342, *p* < 0.0001) on ipsilateral paw withdrawal threshold at 60 min following drug administration on PSD 8. Tukey’s post hoc test revealed a significantly lower paw withdrawal threshold in incision-vehicle rats than in sham-vehicle rats (*p* < 0.001). Tukey’s post hoc test revealed no significant differences between incision-CBD (3 mg/kg) and incision-vehicle rats, between incision-CBD (10 mg/kg) and incision-vehicle rats, or between incision-CBD (30 mg/kg) and incision-vehicle rats (*p* > 0.05), indicating that CBD does not significantly attenuate incision-related mechanical hypersensitivity in the ipsilateral hind paw 60 min post-administration, as shown in Figure 2C.

One-way ANOVA revealed a significant effect of treatment (F_(4, 40)_ = 6.720, *p* < 0.0001) on the contralateral paw withdrawal threshold at 60 min following drug administration on PSD 8. Tukey’s post hoc test revealed a significantly lower paw withdrawal threshold in incision-vehicle rats than in sham-vehicle rats (*p* < 0.01). Tukey’s post hoc test revealed no significant differences between incision-CBD (3 mg/kg) and incision-vehicle rats, between incision-CBD (10 mg/kg) and incision-vehicle rats, or between incision-CBD (30 mg/kg) and incision-vehicle rats (*p* > 0.05), indicating that CBD does not significantly attenuate incision-related mechanical hypersensitivity in the contralateral hind paw 60 min post-administration, as shown in Figure 2D.

### 2.3. CBD Does Not Alter Levels of Endocannabinoids or N-Acylethanolamines in the Plasma, Spinal Cord or Discrete Brain Regions

The levels of *N*-acylethanolamines (PEA and OEA) and endocannabinoids (2-AG and AEA) in the plasma and discrete brain regions were analysed 90 min after vehicle or CBD administration.

**Figure 3:**
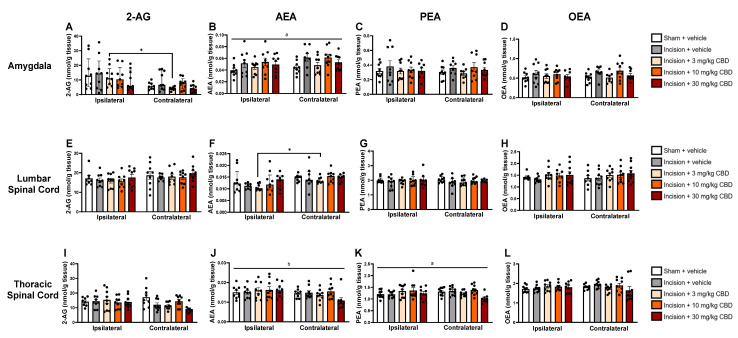
Levels of endocannabinoids and *N*-acylethanolamines in the ipsilateral and contralateral amygdala (**A**–**D**), lumbar spinal cord (**E**–**H**), and thoracic spinal cord (**I**–**L**), 90 min after vehicle or CBD administration. Data are expressed as mean ± S.E.M (*n* = 8–9 per group). a = significant effect of treatment; b = significant effect of side in ANOVA. * *p* < 0.05, ipsilateral vs. contralateral CBD (3 mg/kg).

Kruskal–Wallis test revealed a significant main effect on the level of 2-AG in the ipsilateral and contralateral amygdala χ^2^(9) = 22.144 (*p* = 0.008). Mann–Whitney U test, followed by Bonferroni–Holm correction, revealed significantly lower 2-AG levels in the contralateral amygdala than in the ipsilateral amygdala in the incision-CBD (3 mg/kg) group only (Figure 3A). Two-way ANOVA revealed a significant effect of treatment on AEA levels in the amygdala (F_(4, 79)_ =2.843, *p* = 0.029) (Figure 3B). Tukey’s post hoc test revealed no significant differences for relevant pair-wise group comparisons.

Kruskal–Wallis test revealed a significant main effect on the levels of AEA in the ipsilateral and contralateral lumbar spinal cord χ^2^(9) = 23.614 (*p* = 0.005). Mann–Whitney U test, followed by Bonferroni–Holm correction, revealed significantly higher AEA levels in the contralateral lumbar spinal cord compared than in the ipsilateral lumbar spinal cord in the incision-CBD (3 mg/kg) group only (Figure 3F).

Two-way ANOVA revealed an effect of side (F_(1, 78)_ = 4.885, *p* = 0.03) on AEA levels in the thoracic spinal cord (Figure 3J). Two-way ANOVA revealed a significant effect of treatment on PEA levels in the thoracic spinal cord (F_(4, 79)_ =2.823, *p* = 0.03) (Figure 3K). Tukey’s post hoc test revealed no significant differences for relevant pair-wise group comparisons.

**Figure 4:**
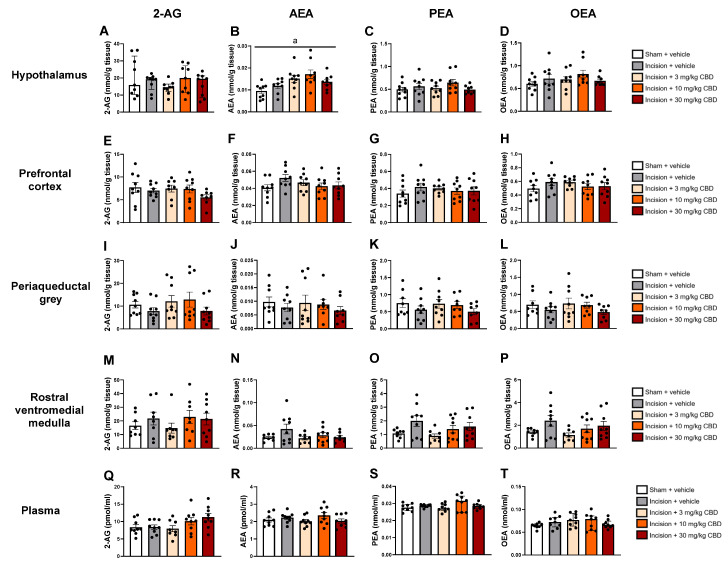
Levels of endocannabinoids and *N*-acylethanolamines in the hypothalamus (**A**–**D**), prefrontal cortex (**E**–**H**), periaqueductal grey (**I**–**L**), rostral ventromedial medulla (**M**–**P**) and plasma (**Q**–**T**) 90 min after vehicle or CBD administration. Data are expressed as mean ± S.E.M (*n* = 8–9 per group). a = significant effect of treatment in ANOVA.

One-way ANOVA revealed a significant effect of treatment (F_(4, 39)_ =4.288, *p* = 0.006) on AEA levels in the hypothalamus 90 min after drug administration on PSD 8. Tukey’s post hoc test revealed no significant differences for relevant pair-wise group comparisons (Figure 4B).

No significant differences in the levels of *N*-acylethanolamines or endocannabinoids were found in the prefrontal cortex (Figure 4E–H), periaqueductal grey (Figure 4I–L), rostral ventromedial medulla (Figure 4M–P), or plasma (Figure 4Q–T).

### 2.4. CBD Does Not Alter Expression of Htr1a, Pparg, or Trpv1 in the Spinal Cord or Discrete Brain Regions

The expression of mRNA encoding *Htr1a*, *Pparg*, and *Trpv1*, genes that encode the 5-HT1A receptor, PPARγ and TRPV1, respectively, 90 min after vehicle or CBD administration was assessed in discrete brain regions using RT-qPCR.

**Figure 5:**
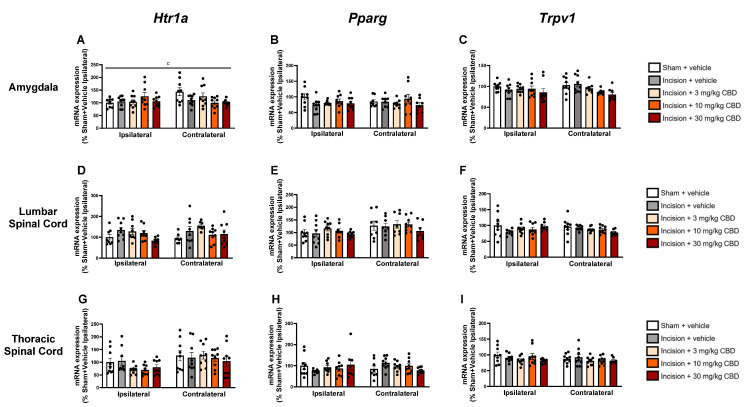
Levels of mRNA encoding *Hrt1a*, *Pparg* and *Trpv1* in the ipsilateral and contralateral amygdala (**A**–**C**), lumbar spinal cord (**D**–**F**), and thoracic spinal cord (**G**–**I**), 90 min after vehicle or CBD administration. Data expressed as mean ± S.E.M (*n* = 8–9 per group). c = treatment × side interaction effect in ANOVA.

Two-way ANOVA revealed a treatment × side interaction effect (F_(4, 78)_ = 2.891, *p* = 0.027) on the expression of mRNA encoding *Htr1a* in the ipsilateral and contralateral amygdala. Tukey’s post hoc test revealed no significant differences for relevant pair-wise group comparisons (Figure 5A).

**Figure 6:**
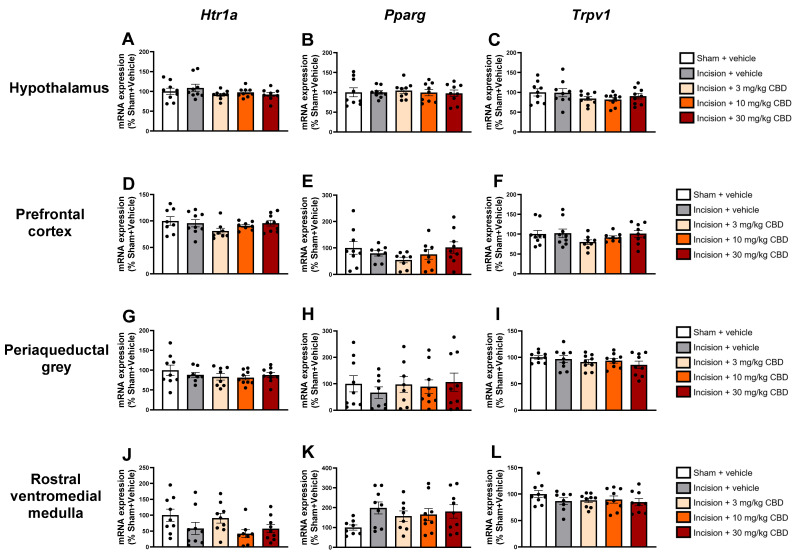
Levels of mRNA encoding *Hrt1a*, *Pparg,* and *Trpv1* in the hypothalamus (**A**–**C**), prefrontal cortex (**D**–**F**), periaqueductal grey (**G**–**I**), and rostral ventromedial medulla (**J**–**L**) 90 min after vehicle or CBD administration. Data expressed as mean ± S.E.M (*n* = 8–9 per group).

No significant differences were found in the expression of *Htr1a*, *Pparg*, or *Trpv1* in the lumbar spinal cord (Figure 5D–F), thoracic spinal cord (Figure 5G–I), hypothalamus (Figure 6A–C), prefrontal cortex (Figure 6D–F), periaqueductal grey (Figure 6G–I), or rostral ventromedial medulla (Figure 6J–L).

## 3. Discussion

These data indicate a robust pain-related behavioural phenotype following dorsum incisional wound creation, as evidenced by the development of mechanical hypersensitivity in both the dorsum and hind paws. The results indicate that mechanical hypersensitivity at 1 cm ipsilateral to the dorsum was attenuated by a single, acute i.p. administration of 3 mg/kg CBD but not by higher CBD doses (10 or 30 mg/kg). CBD did not attenuate secondary mechanical hypersensitivity at 2 cm contralateral to the incision or in the ipsilateral (left) and contralateral (right) hind paws. CBD did not alter the levels of endocannabinoids or *N*-acylethanolamines in the plasma, spinal cord, or brain, or alter the expression of *Htr1a*, *Pparg,* or *Trpv1* in key brain regions related to nociception.

CBD had antinociceptive effects at 1 cm ipsilateral to the incision on the dorsum, the most proximal testing site to the incision, but not at 2 cm contralateral to the incision on the dorsum, or in the hind paws. The mechanism through which CBD exerts its antinociceptive effects may explain why its effects were restricted to the attenuation of primary mechanical hypersensitivity only and not secondary hypersensitivity. CBD has anti-inflammatory effects; a CBD-containing hydrogel can reduce tumour necrosis factor-alpha (TNF)-α and interleukin (IL)-6 and IL-1β levels when applied to cells compared to the hydrogel control, while CBD decreases IL-33 expression in the wounds of mice with a dorsum full-thickness wound [26,27]. The anti-inflammatory effects of CBD at the incision site may attenuate primary hypersensitivity at 1 cm ipsilateral to the wound but not affect secondary hypersensitivity at 2 cm contralateral to the incision on the dorsum, or in the hind paws. The secondary hypersensitivity may be mediated by other mechanisms (e.g., central sensitisation). However, CBD administered directly into the rostral anterior cingulate cortex attenuates primary hypersensitivity in the paw incision model, indicating that supraspinal mechanisms may also contribute to the antinociceptive effects of CBD in the back hairy skin incision model [18]. CBD has also been shown to have the ability to modulate ascending and descending nociceptive pathways in areas such as the PAG, providing further evidence for supraspinal-mediated antinociceptive effects [28]. While this study focused on the effects of CBD on the endocannabinoid system and the expression of genes encoding receptor targets in the CNS, a limitation of the current study is that peripheral or site-specific effects at the wound were not investigated. Future work should investigate the effects of systemic CBD on inflammatory markers at the site of the wound and assess the antinociceptive and anti-inflammatory effects of local CBD administration in this wound model.

It is also possible that CBD did not attenuate mechanical hypersensitivity at 2 cm contralateral to the wound or in the hind paws due to its dose-dependent effects, which have been observed previously. Dose-dependent effects of CBD have been reported clinically, with a low dose (200 mg) of CBD increasing pain threshold, and higher doses (400 and 800 mg) decreasing pain threshold compared to placebo [29]. Biphasic and dose-dependent effects have also been reported clinically for cannabinoids in pain conditions [30]. An inverted U-shaped (or bell-shaped) dose–response curve has been clinically demonstrated for the anxiolytic effects of CBD in a simulated public speaking test. A 300 mg oral CBD dose significantly reduced anxiety, but lower (150 mg) and higher (600 mg) doses had no significant effect on anxiety compared with placebo [31]. This bell-shaped dose–response curve for CBD has also been observed in a preclinical paw incision model. Genaro et al. (2017) found that 3 and 10 mg/kg i.p. doses of CBD significantly increased withdrawal thresholds following paw incision 60 min after administration, but 0.3, 1, and 30 mg/kg CBD doses had no significant effect on paw withdrawal thresholds [18]. This study provided rationale for the time of behavioural testing and the doses used in the present study. In the dorsum incision model, 10 and 30 mg/kg doses had no effect on mechanical hypersensitivity; however, doses lower than the effective 3 mg/kg dose were not assessed, which is a limitation of the present study. Future studies should examine the effects of lower doses of CBD (e.g., 1 or 2 mg/kg) on mechanical hypersensitivity to determine whether these doses are effective at attenuating mechanical hypersensitivity in both the dorsum and paws. One hypothesis for the bell-shaped dose–response curve for CBD is the differential activation of receptors at ‘low’ and ‘high’ doses of CBD, which produce conflicting behavioural effects [31]. Other reasons for the dose-dependent effects of CBD include pharmacokinetic alterations, metabolic saturation and/or receptor desensitisation that can occur at higher doses and have been shown to be dose-dependent [32]. Another potential limitation is that the effects of CBD on locomotor activity were not assessed in the present study. However, there is evidence that a single acute dose of CBD (10 mg/kg, i.p.) does not significantly alter locomotor activity 60 min after administration in male rats, the time point that nociceptive behaviour was assessed in the present study [33]. Furthermore, there is evidence that a CBD (3 mg/kg) does not alter locomotor activity in male rats 20 min after administration [34]. Chronic studies investigating the effect of repeated CBD administration in this model should also be conducted, as several days of CBD administration may be required to observe the antinociceptive effects of CBD. For example, CBD significantly increased withdrawal thresholds in the rat SNI model after 7 days of daily subcutaneous administration, an effect that was not evident after five days of daily CBD administration [16].

CBD did not significantly alter the levels of *N*-acylethanolamines (PEA and OEA) or endocannabinoids (2-AG and AEA) in the plasma, spinal cord, or discrete brain regions related to nociception and pain processing. A significant increase in AEA levels was observed in the ipsilateral vs. contralateral amygdala and a decrease in 2-AG levels in the ipsilateral vs. contralateral lumbar spinal cord in the 3 mg/kg CBD group. This result suggests potential dose-specific, region-specific and side-specific effects of CBD on the endocannabinoid system. A limitation of the present study is that CBD levels in plasma and tissue were not assessed, which may limit the interpretation of the molecular analysis, but was outside the scope of the present study. CBD has activity at targets both internal and external to the endocannabinoid system, which has been extensively reviewed [35]. In the endocannabinoid system, although CBD has poor affinity for the orthosteric site on cannabinoid receptors, it has activity at the allosteric binding sites of these receptors [36]. By weakly inhibiting FAAH, CBD can also indirectly target the endocannabinoid system [37]. However, CBD-induced increases in the FAAH substrates AEA, PEA, and OEA were not observed in the present study. The absence of CBD-induced alterations does not rule out a role for the endocannabinoid system in the antinociceptive effects of CBD through mechanisms such as allosteric modulation of cannabinoid receptors or antagonist activity at GPR55 [38]. Furthermore, the time of euthanasia post-CBD administration may not have been optimal for detecting CBD-induced alterations in the levels of endocannabinoids and *N*-acylethanolamines. The focus of the present study was on behavioural outcomes; however, future studies could investigate the temporal profile of CBD-induced alterations in the endocannabinoid system at several time points after CBD administration.

CBD did not alter the expression of mRNA encoding *Pparg*, *Htr1a*, and *Trpv1*. The time point of euthanasia (90 min after CBD administration, 30 min after the commencement of pain-related behavioural testing) may not have been optimal for assessing increases in the levels of mRNA encoding these genes using RT-qPCR. Alterations in CBD-associated target expression in the lumbar spinal cord have been observed in the monoiodoacetate model of osteoarthritis, where the expression of *Trpv1* and *Trpa1* was reduced in the lumbar spinal cord of CBD-treated rats compared to vehicle-treated controls; however, the expression of mRNA encoding these genes was assessed 5 h after CBD administration [39]. A limitation is that it is possible that alterations in the protein expression or activity of these receptors may occur following CBD administration, which were not assessed in this study. Daily CBD administration for 3 days attenuated CCI-induced increases in the expression of TRPV1 and CB_1_ receptors in several brain regions, including the basolateral amygdala, anterior insular cortex, and ventral hippocampus [17]. It is possible that the time point in this study was too early to assess CBD-induced alterations in the mRNA expression of these targets. Other differences between these studies include the model (incisional pain vs. neuropathic pain), dosing regimen (single acute vs. daily for 3 days), and assessment method (RT-qPCR vs. immunohistochemistry), which may further account for the lack of alterations in the expression of these targets observed in the present study. CBD has a T_max_ of between 60 and 120 min and a half-life of 289–347 min in rodent brain tissue following i.p. administration, so it is possible that this time point was too early to observe alterations in gene expression in the brain [40]. The possibility of delayed or biphasic molecular responses to CBD may also have contributed to the lack of alterations in gene expression observed in this study The results of the present study support the contention that the antinociceptive effects of CBD in incisional wound-related pain are independent of alterations in the expression of genes encoding *Pparg*, *Htr1a*, and *Trpv1* 90 min after drug administration under the conditions tested herein, but as discussed, time point-related limitations may account for the lack of observed alterations. Furthermore, the pharmacology of CBD at its numerous targets includes allosteric modulation, receptor desensitisation, and post-translational effects that may not be reflected at the transcriptional level investigated in the present study. Antagonist studies in rat pain models have indicated that the antinociceptive effects of CBD are at least partially mediated by these receptors; however, future studies should confirm whether this hypothesis is true for incisional wound-related pain [11,12,16,22,25].

This experiment was conducted using male rats only, which represents a significant limitation. However, the decision to use male rats herein was based on evidence from a recent study in our laboratory [41]. This study revealed sex differences following dorsum incision, with a more robust pain phenotype in males post-incision. Primary hypersensitivity persisted to post-incision day 14 in males but only to day seven in females. The use of only male rats limits the translational relevance of these findings to females, given the reported sex differences in the endocannabinoid system and cannabinoid pharmacology clinically [42,43]. Sex differences in the effective CBD dose have also been observed, where the maximum antinociceptive effect of CBD in males in the tail-flick test was found to be at a dose 100 times higher than that of females during the late dioestrus phase [19]. However, a 3 mg/kg dose of CBD partially attenuated mechanical hypersensitivity in both male and female rats following paw incision. These data suggest that sex differences in antinociceptive effects may occur in incisional wound models, but these sex differences may be modality-dependent (e.g., sex differences in CBD’s effects on heat but not mechanical hypersensitivity). For maximum clinical translatability of the results obtained, the experiment in the back hairy skin incision model should be repeated in female rats to observe whether the effects observed are relevant to the female sex.

These results indicate dose- and site-specific antinociceptive effects of CBD in a rat model of incisional wound-related pain, providing preclinical evidence to support the contention that CBD may have therapeutic potential for alleviating incisional wound-related pain. CBD did not alter endocannabinoid or *N*-acylethanolamine levels or the expression of mRNA encoding *Pparg*, *Htr1a*, or *Trpv1* under the conditions tested herein. However, the antinociceptive effects of CBD may still be mediated by the agonist activity of CBD at these receptors without altering gene expression. Overall, these data provide a basis for further investigation to elucidate the putative mechanisms of CBD by which attenuation of pain-related behaviour in the dorsum occurs. These results also indicate that the potential antinociceptive effects of other phytocannabinoids should be investigated in this model of incisional wound-related pain.

## 4. Materials and Methods

### 4.1. Animals

The experiment was carried out on 45 male Sprague Dawley rats (6–7 weeks, 160–200 g on arrival; Charles River, Margate, UK). Rats were maintained under standard 12:12 h light/dark cycle (lights on from 07:00 to 19:00 h) in a temperature- (21 ± 2 °C) and humidity-controlled (45–55%) room throughout the study. The light intensity in the room was approximately 200 lx and approximately 30 lx at the cage floor level. Food (14% Harlan Teklad 2014 Maintenance Diet, Envigo, Loughborough, UK) and water were available ad libitum. Rats were housed in plastic cages (45 × 20 × 20 cm^3^) containing ABP3 poplar bedding (Datesand Ltd., Stockport, UK), Sizzle-Pet^®^ material (LBS Biotechnology, Horley, UK), and a red plastic tube for standard environmental enrichment. After habituation for at least five days, the animals were singly housed for the duration of the experiment.

The experimental procedures were approved by the Animal Care and Research Ethics Committee of the University of Galway (Approval Code: 20-Sept-01; 2 December 2020). These experiments were completed under a licence from the Health Products Regulatory Authority in the Republic of Ireland, in accordance with the EU Directive 2010/63 (Project authorisation number: AE19125/P106). These studies were designed and reported in accordance with the ARRIVE 2.0 guidelines [44].

### 4.2. Experimental Design

**Figure 7:**
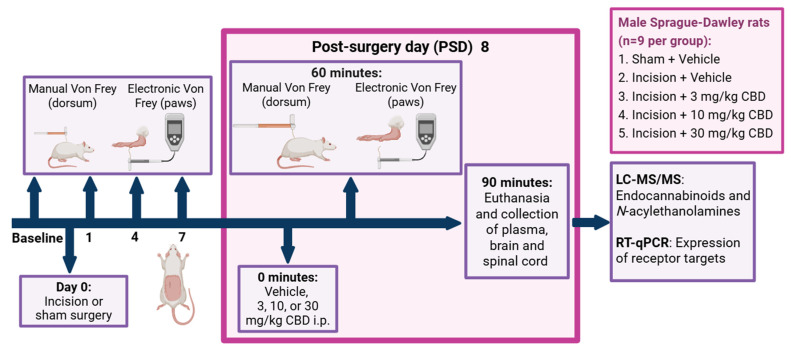
Experimental design for the investigation of the effect of CBD on nociceptive behaviour in the back hairy skin incision model, 8 days post-sham or incision surgery. PSD: day(s) post-back hairy skin incision or sham procedure; i.p., intraperitoneal administration. Created in BioRender. Redmond, MC. (2025) https://BioRender.com/92d01ns.

The experimental design is shown in Figure 7. The research question, experimental design and data analysis plan were prepared prior to the study but was not registered. After at least 5 days following the commencement of single housing, baseline mechanical sensitivity testing was performed 5 and 3 days prior to back incision or sham procedure using manual and electronic von Frey tests on the dorsum and hind paws, respectively. The dorsum of each rat was shaved under brief isoflurane anaesthesia 24 h before the first baseline test. The dorsum was subsequently re-shaved while the animals were awake and moving 24 h prior to each behavioural testing session. After baseline testing, animals were pseudo-randomly assigned to either the sham (*n* = 9) or incision (*n* = 36) groups to ensure no significant differences in weight or average scores for baseline mechanical withdrawal thresholds between groups. Sample size per group was calculated based on power analysis for the von Frey test as our primary outcome measure, based on the scientific literature where similar models and outcome measures were used.

Manual and electronic von Frey tests were performed on the dorsum and hind paws, respectively, on PSDs 1, 4, and 7. Following behavioural testing on PSD 7, incision rats were further pseudo-randomly assigned to receive either vehicle or CBD (3, 10, or 30 mg/kg) to ensure no significant differences in weight or mechanical withdrawal thresholds between incision groups prior to drug administration. On PSD 8, rats received an i.p. injection of vehicle (Cremophor–ethanol–saline 1:1:18), 3 mg/kg CBD, 10 mg/kg CBD, or 30 mg/kg CBD in an injection volume of 2 mL/kg and were subjected to mechanical sensitivity testing 60 min post-drug administration. The researcher who performed the behavioural testing was female and blinded to the drug treatment. Rats were euthanised by live decapitation 90 min after drug administration and 30 min after the commencement of pain-related behavioural testing. The experimental unit was one rat.

### 4.3. Shaving and Marking of the Dorsum

Initial shaving and marking of the dorsum were completed under isoflurane anaesthesia 24 h before the first baseline test. Animals underwent anaesthesia induction in a plexiglass chamber with 5% isoflurane (Iso-Vet^®^) (Chanelle Pharma, Galway, Ireland) at 0.8 L/min O_2_ for 3–5 min. Anaesthesia was maintained at 1.5–2.5% isoflurane in 0.8 L/min O_2_ for the duration of the procedure through a nose cone. Under anaesthesia, the dorsum was shaved from the lower thoracic region to the top of the hipbone line. The location of the incision and the two testing sites on the dorsum (1 cm ipsilateral to the incision and 2 cm contralateral to the incision) were marked with a permanent marker. Following recovery from anaesthesia, the rats were placed individually into a recovery chamber maintained at 26 °C for 30 min before being returned to their home cage.

After the initial shaving and marking under anaesthesia, subsequent shaving and marking of the dorsum were completed in awake rats while being held by the researcher. The animals were habituated to handling and the sound of the shaver throughout the habituation period.

### 4.4. Rat Back Hairy Skin Incision or Sham Procedure

The incision procedure was performed as previously described [21]. Anaesthesia was induced and maintained as described above for the shaving and marking procedures. Once anaesthetised, Vidisic (0.2% *w*/*w*) eye gel (Bausch & Lomb Ireland Ltd., Dublin, Ireland) was placed in the eyes to maintain moisture. The shaved area of the dorsum was re-shaved and disinfected with a chlorhexidine solution. A 1.2 cm incision was made using a no. 10 surgical blade 0.5 cm posterior to the L4 transverse process and 0.5 cm to the left of the midline. Blunt dissection of the skin away from the underlying fascia was performed using blunt scissors (Fine Science Tools, Heidelberg, Germany) at a radius of 0.5 cm around the wound. The area was cleaned with sterile saline and cotton buds. The wound was closed using two sutures with non-absorbable 4-0 Mersilk sutures (Johnson and Johnson, Dublin, Ireland). The testing sites on the dorsum were re-marked under aseptic conditions. For the sham procedure, rats were exposed to the same anaesthetic conditions and marking of the dorsum, but no incision, blunt dissection, or suturing was performed. The duration of each sham/incision procedure was–10–15 min from the time of anaesthesia induction. After the incision or sham procedure, the rats were singly placed in a recovery chamber maintained at 26 °C for a minimum of 30 min before being returned to the home cage.

General distress scoring sheets were used to assess health and welfare twice within the first 24 h after surgery, daily for the first 5 days post-surgery, and every second day thereafter. A specific distress scoring table was used for the daily monitoring of potential adverse effects related to the back incision procedure. Humane endpoints were a score greater than 13 in the distress scoring sheet, loss of body weight of more than 20% of the pre-procedural weight, removal of sutures resulting in the wound becoming compromised, or if an animal was found to exhibit a complete lack of activity or eyes closed when stimulated or gasping for breath. No animals met a humane endpoint or were removed from this study.

### 4.5. Pain-Related Behavioural Testing

Pain-related behavioural testing was performed on both the dorsum (1 cm ipsilateral and 2 cm contralateral to the dorsum incision) and hind paws using manual and electronic von Frey testing, respectively. The light intensity at the arena level was set to 40 lx. Testing was completed at approximately the same time of the day in a quiet, temperature-controlled room.

#### 4.5.1. Manual Von Frey

The rats were habituated for 30 min prior to testing in the testing arena. The testing arenas consisted of curved stainless-steel enclosures (30 × 10 × 8 cm^3^) placed on top of a wire mesh base with open segments to facilitate the application of von Frey filaments to the dorsum. A Perspex^®^ gate located at one end of the arena facilitated the introduction and removal of rats from each arena. Manual von Frey was performed using nylon von Frey filaments (Touch Test Sensory Evaluator #58011, Stoelting, Wood Dale, IL, USA) ranging from 0.6 g to 15 g. A maximum filament weight of 15 g was used to prevent potential local irritation due to the repeated application of filaments heavier than 15 g [21].

Rats were first stimulated at the 2 cm contralateral testing site with the lowest weight filament (0.6 g post-incision/sham procedure and 2 g for baseline testing). The von Frey filament was pressed against the skin until it bent four times per filament at a frequency of approximately once every 5 s. The response to each filament was recorded. A positive response included licking or biting at the site of application, shaking, shuddering, rapid turning, vocalisation, or robust contraction of the skin and subdermal muscles under the testing location.

Following the application of the lowest weight filament to the 2 cm contralateral testing site, the 2 cm contralateral testing site of rats in adjacent and subsequent arenas was tested using the same filament. Following the application of the lowest weight filament to the 2 cm contralateral testing site of all rats in the arena, the 1 cm ipsilateral testing site of each rat was stimulated with the same weight filament. This procedure was repeated with increasing filament weights until the mechanical response threshold was reached at each testing site (2 cm contralateral and 1 cm ipsilateral to the wound). Animals were assigned a mechanical withdrawal threshold of 15.1 g if they did not respond 4/4 times to the maximum filament weight of 15 g.

#### 4.5.2. Electronic Von Frey

The electronic von Frey test began 5 min after the completion of the manual von Frey test on the dorsum in the same testing arena. An electronic von Frey anesthesiometer (IITC Life Science Inc., Woodland Hills, CA, USA) was used to assess mechanical hypersensitivity in both the ipsilateral (left) and contralateral (right) hind paws. The IITC aesthesiometer used 0.8 mm rigid, non-hygroscopic polypropylene tips. Three applications were used to evaluate the mechanical sensitivity of each paw. The left (ipsilateral) and right (contralateral) paws were alternated at intervals of at least 5 min between paws [45,46].

Starting with the contralateral paw, the filament was applied perpendicular to the flat pad of the hind paw with a continuous and steady force until a positive withdrawal response was observed. A positive result was recorded if flinching, licking, or withdrawal of the paw occurred in response to the force applied to the filament. The applied force (in grams) was recorded. The withdrawal threshold of the contralateral hind paw was then tested on the rats in the subsequent arenas. Following the completion of the first trial on the contralateral paw of each rat, the ipsilateral paw was stimulated. This procedure was repeated twice more to obtain three readings per paw. The arenas and mesh bases were cleaned with IMS (70% *v*/*v*) between sessions. The mechanical withdrawal threshold for each paw was calculated by taking the average response (in grams) across three trials per paw.

### 4.6. Drug Preparation

CBD (API) was gifted by Benuvia Operations, LLC (Round Rock, TX, USA) as powder. CBD was dissolved in a vehicle (1:1:18 ethanol–Cremophor–saline (0.9% NaCl)) solution and administered intraperitoneally at a volume of 2 mL/kg. CBD was administered at three different doses on PSD 8:3, 10, and 30 mg/kg. Vehicle-injected rats received an i.p. injection of vehicle solution (1:1:18 ethanol–Cremophor–saline) at a volume of 2 mL/kg. The sham group received vehicle only.

### 4.7. Tissue Collection

Following live decapitation on PSD 8, the spinal cord and brain were gross-dissected, snap-frozen, and stored at −80 °C. Live decapitation was used as the method of euthanasia due to the effect of anaesthesia on levels of endocannabinoids [47,48]. Trunk blood was collected in 10 mL EDTA-coated tubes (Becton Dickinson, Plymouth, UK) and centrifuged at 3420× *g* for 15 min at 4 °C (Eppendorf Centrifuge 5810 R, Hamburg, Germany). Clear, straw-coloured plasma was collected, snap-frozen, and stored at −80 °C for analysis.

### 4.8. Liquid Chromatography/Tandem Mass Spectrometry (LC-MS/MS)

The protocols were similar to those previously described in our laboratory, with minor modifications [49,50,51]. Frozen brain or spinal cord samples were placed on ice for further processing. 200 ul of ice-cold acetonitrile (ACN) containing deuterated internal standards (2.5 ng d4-OEA, 2.5 ng d4-PEA, 50 ng d8-2-AG and 2.5 ng d8-AEA; Cayman Chemicals, Cambridge Biosciences, Cambridge, UK) was added to each sample. 75 μL of ice-cold ACN was added to each sample to obtain a final volume of 275 μL per sample. Samples were sonicated using an ultrasonic homogeniser (SFX150 Cell Disruptor, Branson Ultrasonics™ Sonifier™, Fisher Scientific, Dublin, Ireland) and centrifuged at 16,000× *g* for 15 min at 4 °C. Then, 40 μL of each sample was added to a HPLC vial. A 10-point standard curve (1:4 dilution) was prepared. Then, 75 μL of ice-cold ACN was added to each tube. A solution (25 μL of a solution containing non-deuterated 250 ng 2-AG, and 25 ng AEA, PEA, OEA) was added to standard 10 and mixed. A 1:4 serial dilution was performed, with 25 μL discarded from the final standard (standard 1) to ensure a volume of 75 μL per tube. 200 μL of ACN containing deuterated internal standards was added to each standard.

For plasma samples, 200 µL aliquots of frozen plasma were gently defrosted over ice and centrifuged at 16,060× *g* for 15 min at 4 °C. Next, 20 μL of 10× deuterated internal standard mix was added to each sample (2.5 ng of OEA-d4, 2.5 ng of PEA-d4, 50 ng of 2-AG-d8 and 2.5 ng of AEA-d8 in 100% ACN). The samples were vortexed and allowed to equilibrate for 10 min on ice. 1 mL of ice-cold ACN containing 0.1% formic acid was added to each sample to precipitate the protein. The samples were then refrigerated at 4 °C for 30 min. Samples were then centrifuged at 16,060 × *g* for 15 min at 4 °C. A filter (Fisherbrand™ non-sterile PTFE Hydrophyl, 25 mm, 0.45 μM Syringe Filter, Fisher Scientific, Dublin, Ireland) was placed over a labelled, opened 5 mL SafeSeal tube (Sarstedt Ltd., Wexford, Ireland) corresponding to each sample. A 2 mL syringe with the plunger/piston removed was attached to each filter by carefully inserting the neck of the syringe into the filter inlet. The pistons from each syringe were saved in a sterile container to assist in pushing the samples through the filter. Next, 1100 μL of the supernatant was removed from the samples without disturbing the pellet at the bottom of the tube and loaded into the syringe Once the supernatant had flowed through the filter and into the 5 mL SafeSeal tube, 700 μL of ACN was loaded into the syringe to displace the supernatant trapped by the dead volume of the filter. Following the addition of ACN, the syringe piston was carefully reinserted into the syringe and pushed down to the neck of the syringe to ensure total displacement of the volume in the syringe into the 5 mL SafeSeal tube. The contents of the 5 mL tube were vortexed. Then, 40 μL of each sample was added to a HPLC vial. A 10-point standard curve was prepared as described above; however, 20 μL of 10× deuterated internal standard mix was added instead of 200 μL of 1× internal standard, as described above.

The mobile phases consisted of (A) HPLC grade water with 0.1% (*v*/*v*) formic acid and (B) ACN with 0.1% (*v*/*v*) formic acid with a flow rate of 0.2 mL/min using a Zorbax^®^ SB C18 column (1.8 μm particle dimension, 50 mm length, 2.1 mm internal diameter, Agilent, Santa Clara, CA, USA). Reverse-phase gradient elution involved 45% B for 1 min, with a linear ramp to 100% B for 4 min. 100% B was maintained until 12 min. At 12.01 min, the gradient returned to the initial conditions, with a further 5 min of re-equilibration before the next sample or standard was injected. The retention times of AEA, 2-AG, PEA, and OEA were 7.7, 8.1, 8.2, and 8.5 min, respectively, under these conditions.

Analyte detection was performed in electrospray-positive ionisation mode on an Agilent 1260 Infinity 2 HPLC system (Agilent Technologies Ltd., Cork, Ireland) coupled to a SCIEX QTRAP 4500 mass spectrometer operated in triple quadrupole mode (SCIEX Ltd., Macclesfield, UK). Each analyte was quantified by determining the peak area of each analyte against the peak area of the internal standard for each sample and standard to obtain a ratio of endogenous analyte/non-deuterated internal standard to deuterated internal standard. The ratio of endogenous analyte to deuterated internal standard could then be compared to the standard curve which contained defined concentrations of both deuterated and nondeuterated internal standards. Ratiometric analysis was performed using Skyline (Skyline v22.2, MacCoss Lab Software, Seattle, WA, USA). The calculated concentrations for each sample were exported to Microsoft Excel. The calculated concentrations were expressed in ng. The calculated concentration was then divided by the weight of the tissue or the volume of the sample. The obtained values were then divided by the molecular weight of the analyte to obtain the final value in nmol of analyte per mL/g of sample. The molecular weights (g/mole) of AEA, 2-AG, PEA, and OEA used for analysis were 348.300549, 379.30055, 300.30055, and 326.300549, respectively.

### 4.9. Real-Time Quantitative Polymerase Chain Reaction (RT-qPCR)

Total RNA was isolated from pelleted samples following LC-MS/MS, according to manufacturer instructions provided by the Macherey-Nagel NucleoSpin RNA isolation kit (Mini Kit for RNA purification; Fisher Scientific, Dublin, Ireland). The concentration, purity, and integrity of the RNA were measured using a DeNovix spectrophotometer (DS-11 Spectrophotometer, DeNovix, Wilmington, DE, USA). The optical density (OD) at 260 nm was used to quantify RNA concentration. The integrity and purity of the RNA were ascertained by measuring the ratios of OD260/280 and OD260/230, where a ratio of 1.8–2.0 was indicative of good quality and purity. Following on from this step, isolated RNA samples were frozen at −80 °C until normalisation of the samples took place prior to cDNA synthesis. cDNA synthesis was completed using a High-Capacity cDNA Reverse Transcription Kit (ThermoFisher Scientific, Dublin, Ireland).

RT-qPCR was used to analyse the cDNA. Plates were analysed using the Applied Biosystems StepOne Plus™ Real-Time PCR System (Biosciences, Dublin, Ireland). TaqMan gene expression assays (Biosciences, Ireland) containing forward and reverse primers and FAM-labelled TaqMan probes (Biosciences, Ireland) were used to quantify the genes of interest. Probes were as follows: Htr1a: Rn00561409_s1; Pparg: Rn00440945_m1; Trpv1: Rn00583117_m1; Actb: Rn00667869_m1. Ct values of the target gene were normalised to the endogenous control gene expression for each sample. ΔΔCt method was used to calculate the relative expression of the target gene to the control gene for each sample. Data are expressed as a percentage of the mean (100%) of the ΔΔCt for the control group (male sham or male sham ipsilateral). The researcher who performed the tissue analysis was blind to the treatment groups.

### 4.10. Statistical Analysis

SPSS statistical software (IBM SPSS Statistics, version 26 for Windows; SPSS Inc., Chicago, IL, USA) was used to analyse all data. The normality and homogeneity of variance of the datasets were checked using the Shapiro–Wilk and Levene’s tests, respectively. Normality and homogeneity were assumed when *p* > 0.05.

Parametric time course behavioural data were analysed using repeated measures analysis of variance with procedure (sham or incision) as the between-subject factor and time as the within-subject factor. For the repeated measures ANOVA, the sphericity of the datasets was ascertained using Mauchly’s Test for Sphericity; if the assumption for sphericity was violated, Greenhouse–Geisser correction was used. Post hoc analysis of parametric time course data was performed using an independent sample t-test with Bonferroni–Holm correction to compare groups at each individual time point. A paired sample t-test with Bonferroni–Holm correction was used for parametric data to assess within-subject pairwise comparisons to baseline.

Non-time course parametric data were analysed using a one-way ANOVA with treatment (sham-vehicle, incision-vehicle, incision-3 mg/kg CBD, incision-10 mg/kg CBD, or incision-30 mg/kg CBD) as the between-subject factor. One-way ANOVA was performed because of the unbalanced study design. For lateralised regions, two-way ANOVA was performed with procedure/treatment (sham-vehicle, incision-vehicle, incision-3 mg/kg CBD, incision-10 mg/kg CBD, or incision-30 mg/kg CBD) and side (ipsilateral or contralateral) as the between-subject factors. Post hoc analysis was carried out following one- or two-way ANOVA using Tukey’s Honest Significant Difference test, where appropriate. The level of significance was set at *p* < 0.05.

For non-time course data, if the variance was not homogeneous and/or not normally distributed, or if the data were ordinal, transformations were applied in the following order: (1) square root of the data values, (2) natural logarithm of the data values, and (3) ranking of the data values to assess whether parametric statistics could be applied. Parametric statistical approaches were still used if the highest standard deviation of the dataset being analysed was less than or equal to two times the smallest standard deviation. If the data were not normally distributed and/or the variance was not homogenous even after transformations had been employed, non-parametric statistical analysis was performed.

For non-time course data, non-parametric data were analysed using the Kruskal–Wallis one-way analysis of variance by rank, followed by post hoc Mann–Whitney U test, with Bonferroni–Holm correction for multiple comparisons. For non-parametric time course data, Friedman’s test with post hoc Mann–Whitney U test, with Bonferroni–Holm correction for between-group pairwise comparisons, or Wilcoxon signed rank test with Bonferroni–Holm correction for within-subject pairwise comparisons was carried out.

In the LC-MS/MS and RT-qPCR datasets, the presence of possible outliers was ascertained by the distribution of data. If a data point fell out of the range of (mean − 2 × standard deviation) to (mean + 2 × standard deviation), it was considered an outlier and excluded from subsequent analyses. Statistical analysis of a dataset was performed for all groups together. All graphs representing the data were created using GraphPad Prism 9.0 (San Diego, CA, USA).

## 5. Conclusions

These results indicate dose- and site-specific antinociceptive effects of CBD in a rat model of incisional wound-related pain, providing preclinical evidence to support the contention that CBD may have therapeutic potential for alleviating incisional wound-related pain. CBD does not alter endocannabinoid or *N*-acylethanolamine levels or alter the expression of mRNA encoding *Pparg*, *Htr1a*, or *Trpv1* under the conditions tested herein. However, the antinociceptive effects of CBD may still be mediated by agonist activity of CBD at these receptors, without altering gene expression. Overall, these data provide a basis for further investigation to elucidate the putative mechanisms of CBD by which attenuation of the pain-related behaviour in the dorsum occurs. These results also indicate that investigation of the potential antinociceptive effects of other phytocannabinoids in this model of incisional wound-related pain is warranted.

## Data Availability

The original contributions presented in this study are included in this article. Further enquiries can be directed to the corresponding author.

## Abbreviations

The following abbreviations are used in this manuscript:

ACN	Acetonitrile
CBD	Cannabidiol
IL	Interleukin
TNF	Tumour necrosis factor
TRP	transient receptor potential
